# Diversity of Filamentous Fungi Associated with Dairy Processing Environments and Spoiled Products in Brazil

**DOI:** 10.3390/foods12010153

**Published:** 2022-12-28

**Authors:** Luana Virgínia Souza, Rafaela da Silva Rodrigues, Andressa Fusieger, Raiane Rodrigues da Silva, Sidney Rodrigues de Jesus Silva, Evandro Martins, Solimar Gonçalves Machado, Cinzia Caggia, Cinzia Lucia Randazzo, Antonio Fernandes de Carvalho

**Affiliations:** 1 InovaLeite—Laboratório de Pesquisa em Leites e Derivados, Departamento de Tecnologia de Alimentos, Universidade Federal de Viçosa, Viçosa 36570-900, MG, Brazil; 2 InsPOA—Laboratório de Inspeção de Produtos de Origem Animal, Departamento de Veterinária, Universidade Federal de Viçosa, Viçosa 36570-900, MG, Brazil; 3 Di3A—Dipartimento di Agricoltura, Alimentazione e Ambiente, Università degli Studi di Catania, 95123 Catania, Italy

**Keywords:** air factory contamination, *Cladosporium*, dairy chain, food safety, spoilage fungi

## Abstract

Few studies have investigated the diversity of spoilage fungi from the dairy production chain in Brazil, despite their importance as spoilage microorganisms. In the present study, 109 filamentous fungi were isolated from various spoiled dairy products and dairy production environments. The isolates were identified through sequencing of the internal transcribed spacer (ITS) region. In spoiled products, *Penicillium* and *Cladosporium* were the most frequent genera of filamentous fungi and were also present in the dairy environment, indicating that they may represent a primary source of contamination. For dairy production environments, the most frequent genera were *Cladosporium*, *Penicillium*, *Aspergillus*, and *Nigrospora*. Four species (*Hypoxylon griseobrunneum*, *Rhinocladiella similis*, *Coniochaeta rosae*, and *Paecilomyces maximus*) were identified for the first time in dairy products or in dairy production environment. Phytopathogenic genera were also detected, such as *Montagnula*, *Clonostachys*, and *Riopa*. One species isolated from the dairy production environment is classified as the pathogenic fungi, *R. similis*. Regarding the phylogeny, 14 different families were observed and most of the fungi belong to the Ascomycota phylum. The understanding of fungal biodiversity in dairy products and environment can support the development of conservation strategies to control food spoilage. This includes the suitable use of preservatives in dairy products, as well as the application of specific cleaning and sanitizing protocols designed for a specific group of target microorganisms.

## 1. Introduction

One of the main global concerns for humans, in regard to food safety and security, is the loss of food through waste and spoilage during production [1]. Food Waste Index report prepared by the United Nations (UNEP) shows that 931 million tons of edible food were wasted globally in 2019, which represents 17% of all food produced worldwide [2]. Currently, one of the main goals of the United Stated Department of Agriculture (USDA) and Environmental Protection Agency (EPA) is to halve global retail and consumer food waste by 2030, as well as reduce food losses in production and supply chains [3].

Concerning food spoilage, food products can be chemically, biochemically, physically, or microbiologically spoiled. Bacteria and/or fungi are the main agents that cause microbial spoilage [4]. Filamentous fungi are estimated to account for 5–10% of all food waste and loss in developing countries [5]. Furthermore, fungal contamination represents a major problem for industry and consumers. Fungi may modify the desired characteristics of products and/or have the ability to produce mycotoxins that are a concern for public health [5,6,7].

Filamentous fungi are ubiquitously found in nature and are also common contaminants of raw foods, such as fruits and vegetables and food products of an animal origin, as well as processed foods, mainly dairy products [7,8]. The food production environment is one of the main sources of contamination, with airborne fungal spores being disseminated by aerosolization throughout the processing plant [8].

Contamination by fungi can lead to the appearance of mycelia, which eventually leads to the deterioration of the product [5]. The presence of black, white, green, pink, or yellow spots can also be associated with the development of fungal colonies and, generally, results in the elimination of the entire product at the industrial or consumer level. Other defects associated with fungal development in food matrices include the production of gas, off-flavors and/or off-odors and changes in texture [9,10].

Regarding the ability of some species of fungi to produce mycotoxins, many species of the *Aspergillus* and *Penicillium* genera are capable of producing aflatoxins, ochratoxins, gliotoxin, fumonisins, sterigmatocystin, patulin, fumigaclavine, roquefortine C, mycophenolic acid, and zearalenone [7,11,12,13]. Although mycotoxins in food are well documented, the effect of mycotoxins on the human body are not fully understood. However, some consequences of ingesting fungal toxins have been reported and include liver carcinomas and renal dysfunction [7]. None of these toxins have been linked to outbreaks of dairy products, but their presence in these products cannot be ruled out.

The Brazilian dairy industry represents a large part of the national economy, with milk production estimated at approximately 25 billion liters in 2021 [14]. The fungi associated with dairy product spoilage can cause significant losses for the dairy industry, and several studies have already addressed contamination in different types of products, such as cheese, yogurt, butter, cream, and milk [6,13,15,16,17,18]. Due to the capacity of certain spoilage fungi to growth in low pH and cold temperature (below 10 °C) environments, fermented dairy products can easily be contaminated. Certain fungi can even tolerate low oxygen concentrations; hence, vacuum-packed cheeses are at risk of contamination [5,17].

Considering the importance of the dairy sector to the Brazilian economy and the negative impact that can be caused by fungal microorganisms, understanding the fungal biodiversity in dairy products and in the production environment is the first step to develop strategies to reduce food waste. Although numerous studies have reported the isolation of fungi from foods [16,17,19,20], there is a lack of information on the characterization of fungi isolated from dairy products in Brazil. Therefore, in this study we aimed to isolate and identify filamentous fungi from dairy processing environments and spoiled dairy products from the Zona da Mata region of Minas Gerais, Brazil.

## 2. Materials and Methods

### 2.1. Isolation of Filamentous Fungi

The diversity of spoilage fungi was evaluated using different “preservative-free” dairy products that were spoiled and from dairy processing environments. The cheese evaluated were Mozzarella (3 samples), Reino (2 samples), Montanhês (3 samples), Parmesan (2 samples), Gorgonzola (1 sample), Provolone (2 samples), Coalho (1 sample), Processed cheese (2 samples), and butter Ghee (2 samples). The samples were obtained from 2 different dairy manufacturers from the state of Minas Gerais, Brazil, and diversity was evaluated over a period of six months.

Isolation of spoilage fungi from dairy products was carried out according to Le Lay et al. [21], with modifications. A contaminated portion of each dairy product was removed with sterile forceps and deposited onto the surface of Dichloran Rose Bengal Chlortetracycline Agar (DRBC; Merck, Darmstadt, Germany), Potato Dextrose Agar (PDA; Merck), and Malt Extract Agar (MEA; Merck), and incubated for 5–7 days at 25 °C. 

Airborne fungal spore collections were taken from the warehouse, cold chamber, ghee storage room, product refrigerators, cheese ripening room, gorgonzola ripening room, processing room, cheese packing room, and brine room, using a passive sampling technique. Petri dishes containing DRBC agar, PDA, and MEA were opened for 30 min in each of the previously mentioned environments of the dairy plant. Fungal collection from equipment and tools including the production tables, cheese containers, cheese packaging, and cheese tank was performed using the swab method [22]. A cotton swab was scrubbed across the surfaces using horizontal, unidirectional, and parallel strokes and then streaked onto Petri dishes containing DRBC, PDA, and MEA media. After this procedure, all the plates were incubated for 5 to 7 days at 25 °C.

After incubation, fungal colonies with distinct morphological characteristics were selected. The selected fungi were purified twice using the same agar medium on which they were isolated and confirmed according to colony uniformity after incubation. For maintenance and subsequent use, a sterile inoculation loop was used to remove a few spores, or a tuft of mycelium from the pure cultures, and inoculated onto Petri dishes and inclined tubes with the defined medium and then incubated for 7 days at 25 °C.

### 2.2. Phenotypic Characterization

The isolates were phenotypically characterized using macro-observations. For macroscopic characterization, fungi were subjected to the inoculum point technique, where a fungal fragment was inoculated onto MEA media at a central point on a Petri dish, sealed and incubated inverted for 7 days at 25 °C. The colony characteristics were observed, including coloring (on both sides), growth rate via colony diameter measurements in millimeters from the reverse side, and observation of presence of pigments and exudates [23]. 

### 2.3. Molecular Identification of Fungal Isolates

#### 2.3.1. Extraction of Fungal DNA

After phenotypic characterization, molecular identification of the isolated fungal strains was performed. The DNA was extracted from mycelial plugs using the Wizard^®^ Genomic DNA Purification kit, according to the manufacturer’s instructions (Promega Madison, WI, USA). The quality and concentration of the extracted DNA was measured using a NanoDrop^TM^ Lite Spectrophotometer (Thermo Scientific, Waltham, MA, USA).

#### 2.3.2. PCR Amplification, DNA Sequencing, and BLAST Identification

For PCR amplification and DNA sequencing, the rDNA internal transcribed spacer (ITS) region (for all isolates) was PCR-amplified using primers ITS4 (5′-TCCTCCGCTTATTGATATGC-3′) and ITS5 (5′- GGAAGTAAAAGTCGTAACAAGG-3′) [24]. PCR amplification and DNA sequencingwere performed in a Thermocycler (MaxyGene^®^ Gradiente Thermal Cycler, Axygen Scientific, Union City, CA, USA).

PCR reactions were performed using a final volume of 25 μL, containing 1X Go Taq^®^ Flexi Buffer [5 X] (Promega); 1.5 mM MgCl_2_ [25 mM]; 0.2 mM of each dNTP [10 mM dNTP] (Promega); 0.2 μM of the ITS4 oligonucleotide; 0.2 μM of the ITS5 oligonucleotide; 1.25 U of Go Taq^®^ DNA Polymerase (Promega) and 20 ng/μL of genomic DNA; and made to volume with autoclaved ultrapure water. A negative control (without the DNA) was included. The PCR protocol utilized had an initial denaturation step at 95 °C for 2 min, followed by 39 cycles at 95 °C for 1 min (denaturation), 50 °C for 1 min (annealing) and 72 °C for 1 min (extension), and a final extension step at 72 °C for 7 min. After amplification, corresponding amplicons were analyzed by electrophoresis on 1% agarose gel (*w*/*v*) for 2 h at a constant voltage of 60 mV in 0.5 X TBE buffer. The gels were stained using GelRed (Biotium Inc., Hayward, CA, USA) and developed using an LPIX transilluminator (Loccus Biotecnologia, São Paulo, Brazil).

PCR products were sequenced by a commercial sequencing service (ACTGene, Porto Alegre, Brazil) and the obtained sequences were edited using MEGA (Molecular Evolutionary Genetics Analysis) software version 11.0.11 (Pennsylvania State University, State College, PA, USA) [25]. Isolate identification was performed using the BLAST (Basic Local Alignment Search Tool, http://www.ncbi.nlm.nih.gov/blast/, accessed on 10 July 2022) against the internal transcribed spacer (ITS) region from fungi type and reference material database of NCBI (National Center for Biotechnology Information). Fungal strains were deposited in the culture collection of InovaLeite (Laboratory of Milk and Dairy Products, Federal University of Viçosa, Viçosa, Minas Gerais, Brazil). Sequences were deposited in GenBank under accession numbers OP584542 to OP584650.

### 2.4. Phylogenetic Analysis

To demonstrate evolutionary relationships between fungal isolates, the ITS sequences from this study, along with other ITS sequences from the National Center for Biotechnology Information (NCBI) Reference Sequence (RefSeq) database, were used. Phylogenetic analysis was performed using MEGA (Molecular Evolutionary Genetics Analysis) software version 11.0.11 (USA) [25]. Sequences were aligned using the MUSCLE algorithm [26]; the phylogeny was inferred using Maximum Likelihood (ML) [27]. The phylogenetic trees were constructed using the Kimura 2-parameter [28] and Tamura 3-parameter [29] substitution models, with a discrete Gamma distribution (+G) with 5 rate categories. Gaps or missing data were treated as partial deletion with a site coverage cut-off value of 95%. Branch support was determined through bootstrapping using 1000 replicates [30]. Trees were viewed and edited using the Interactive Tree Of Life (iTOL v.6.5.4 web-based tool (Germany) [31].

## 3. Results

### 3.1. Isolation of Filamentous Fungi

**Figure 1:**
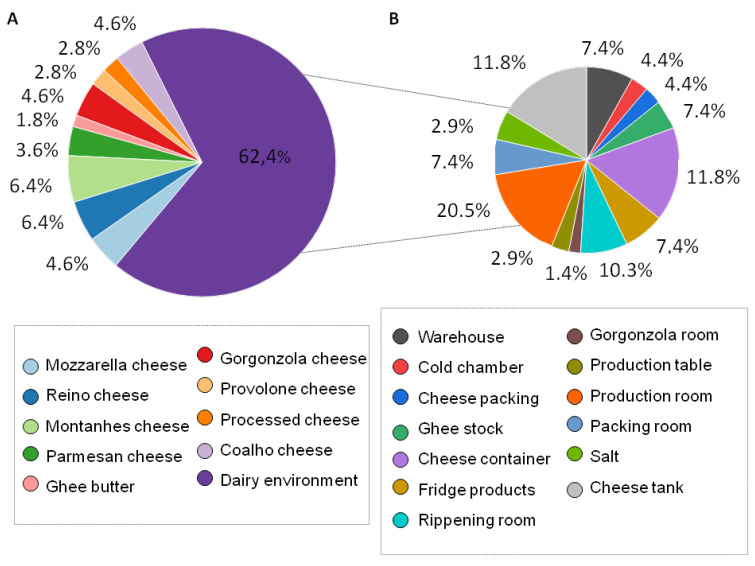
Percentage of filamentous fungi isolated from spoiled dairy products (**A**) and dairy processing environment, equipment, and tools (**B**).

A total of 109 filamentous fungi were isolated, with 37.6% being isolated from spoiled dairy products and 62.4% from the dairy plant environment (Figure 1). Fungi isolated from dairy products ranged from 1.8% to 6.4% of the total number of isolated fungi, with Montanhês and Reino cheese having the highest occurrence (6.4%) and Ghee butter with the lowest percentage (1.8%).

The percentage of the biodiversity fungi isolated from the environment had a varied distribution, with the production room (20.5%), cheese containers, and cheese tank surfaces having the highest proportion (11.8%) of filamentous fungi and the gorgonzola ripening room having the lowest proportion (1.4%).

### 3.2. Identification of Fungal Isolates

A total of 85 isolates (59.44%) were identified to the genus level through ITS amplification and sequencing. From these, 16 different genera were identified as: *Penicillium* (36 isolates), *Cladosporium* (23 isolates), *Nigrospora* (5 isolates), *Riopa* (5 isolates), *Aspergillus* (4 isolates), *Hipoxylon* (2 isolates), *Fusarium* (1 isolate), *Montagnula* (1 isolate), *Clonostachys* (1 isolate), *Phaeosphaeria* (1 isolate), *Rhinocladiella* (1 isolate), *Coniochaeta* (1 isolate), *Trichoderma* (1 isolate), *Paecilomyces* (1 isolate), *Didymella* (1 isolate), and *Bipolaris* (1 isolate). A total of 24 isolates (16.78%) could not be identified at genus level (Supplementary Table S1). A further 34 fungal isolates (23.78%) could not be amplified using ITS primers. Despite the variety of fungi identified, it is very difficult to distinguish between them, due to the high similarity in morphological and biological characteristics between the different groups. The ITS region that was used in this study is the most used marker for filamentous fungi identification, considering that most fungi have this specific region [18].

Regarding the identified genera, at least one fungus was found in each of the 8 evaluated products (Table 1). The *Penicillium* genus was the most prevalent (22.94%) and was found in all spoiled dairy products, except processed cheese. The *Phaeosphaeria* and *Fusarium* genera were found (0.92%) in only one type of product, coalho and montanhês cheese, respectively. The *Cladosporium* genus was found (5.5%) in three types of cheese: montanhês, coalho, and processed cheese. Only these 4 genera (*Penicillium*, *Cladosporium*, *Phaeosphaeria*, and *Fusarium*) appeared in dairy products. The other genera found in this study were isolated from the environment, equipment, and tools (Table 2).

At least one fungal genus was isolated (Table 2) from each dairy environment (air, equipment, and tools). The *Cladosporium* genus was the most frequent (15.60%), being present in most of the sampled environments, followed by *Penicillium* (10.09%), *Nigrospora* (4.58%) and *Riopa* (4.58%). Unidentified genera were found in 14.68% of the sampled environments. A greater diversity of genera was found in the production room (8 genera), followed by the cheese tanks (6 genera). Only one fungal genus was found in the ripening gorgonzola room and production tables, *Trichoderma* and *Cladosporium*, respectively.

*Cladosporium* and *Penicillium*, which were the most frequent in the environment, were also detected in dairy products (Table 1 and Table 2). *Fusarium* and *Phaeosphaeria*, found in the dairy products, were not isolated from the dairy environments.

At the species level, 4 isolates were identified (Table 3). The species identified through BLAST of the NCBI database were filtered according to the material type selection criteria.

**Figure 2:**
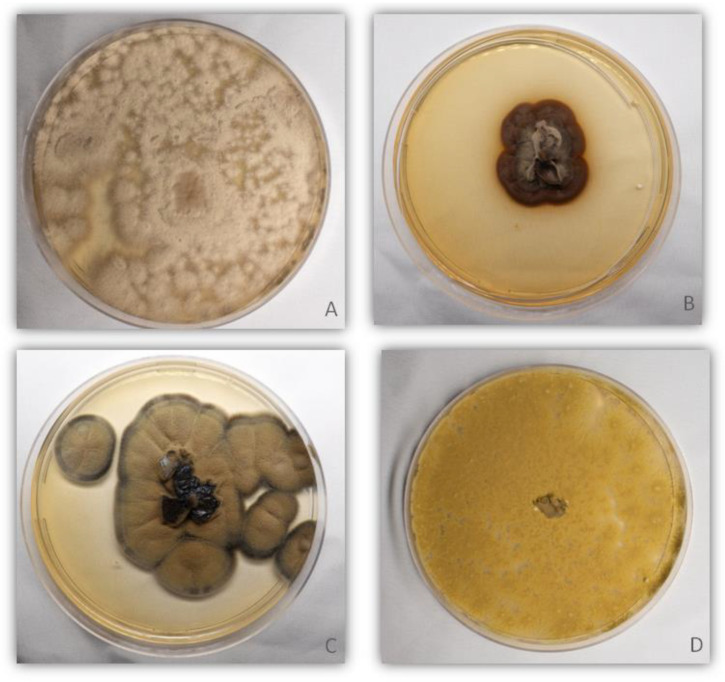
Morphological characteristics of the isolates identified at the species level: (**A**) *H. griseobrunneum*, (**B**) *R. similis*, (**C**) *C.rosae*, and (**D**) *P. maximus*.

In addition to molecular identification, the phenotypic characteristics of the isolates were also considered very similar to the accessioned species previously deposited in NCBI, and the morphological characteristics (Figure 2) were similar to images available in the literature that described the species of *H. griseobrunneum* [32], *R. similis* [33], *C. rosae* [34], and *P. maximus* [35].

### 3.3. Phylogenetic Analysis

Among the 109 isolates, 85 isolates were identified to at least the genus level. Phylogenetic analysis was conducted using the isolated samples’ ITS sequences and with ITS reference sequences from the NCBI database (Supplementary Figure S1). The 85 isolates belonged to six orders (Eurotiales, Xylariales, Coniochaetales, Hypocreales, Chaetothyriales, Cladosporiales, Pleosporales, and Polyporales) and 14 fungal families (Aspergillaceae, Thermoascaceae, Apiosporaceae, Hypoxylaceae, Coniochaetaceae, Nectriaceae, Bionectriaceae, Hypocreaceae, Herpotrichiellaceae, Cladosporiaceae, Didymosphaeriaceae, Didymellaceae, Phaeosphaeriaceae, Pleosporaceae, and Phanerochaetaceae). In addition, the isolates belonging to the genus *Riopa* were the only isolates found for the phylum Basidiomycota, while the other isolates belonged to the phylum Ascomycota.

**Figure 3:**
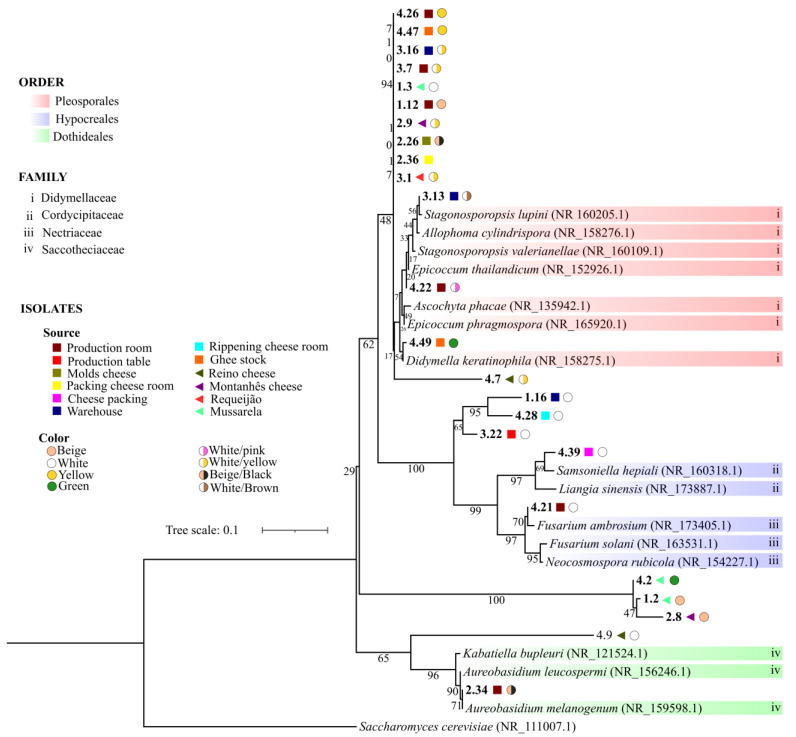
Phylogenetic analysis of not identified isolates.

It was not possible to identify 24 sequenced isolates, and eight of them had no similarity with any of the ITS reference sequences. However, 11 had query coverage and percent identity above 95% with three species of different genera: *Epicoccum phragmospora*, *Didymella keratinophila*, and *Ascochyta phacae*, all belonging to the Pleosporales order, and Didymellaceae family. An inferred phylogeny, using the unidentified isolates and reference sequences with greater similarity from the NCBI database, showed that these 11 isolates; the fungi type; two other isolates with similarity to other species, but of the same order and family (4.22 and 3.13); and one with no similarity to any reference sequence (4.7) clustered together. However, no pattern in the source of isolation and fungal color was observed (Figure 3). More information about these isolates is described in the Supplementary Table S1.

Phylogeny based on ITS region nucleotide sequences, inferred using Maximum Likelihood and Tamura 3-parameter substitution model [29]. The tree with the highest log likelihood is shown. A discrete Gamma distribution was used to model evolutionary rate differences among sites. All positions with less than 95% site coverage were eliminated (partial deletion option). There was a total of 352 positions in the final dataset. Numbers at branches indicate percentages of bootstrap values obtained from 1000 replicates. *Saccharomyces cerevisiae* was used as an out group. The codes 4.26, 4.47, 3.16, 3.7, 1.3, 1.12, 2.9, 2.26, 2.36, 3.1, 3.13, 4.22, 4.49, 4.7, 1.16, 4.28, 3.22, 3.22, 4.39 4.21, 4.2, 1.2, 2.8, and 2.34 are the isolates that were not identified. 

## 4. Discussion

The current results highlight the great diversity of fungi that are associated with dairy products and dairy processing environments in the Zona da Mata region of Minas Gerais (Brazil), identifying a total of 16 genera. It is worth mentioning that the majority of fungi was isolated from the environment; however, a considerable amount of fungi was still isolated from spoiled dairy products, such as semi-hard (Coalho, Montanhês, Reino, and Provolone) and hard cheese (parmesan cheese). The designation of cheese as semi-hard or hard is related to the moisture content, where cheeses with moisture content between 49% to 56% and 54% to 69% are classified as hard and semi-hard, respectively [36]. These cheeses can be ripened for a specific period until reaching the desired and particular characteristics of each type of cheese. Throughout storage, several factors can influence cheese spoilage, if conservation techniques are not applied effectively [37]. Even though these types of cheeses are considered as having a relatively long shelf life, they can still be spoiled by filamentous fungi in storage, as highlighted in this study.

Garnier et al. [17] also identified filamentous fungi from spoiled dairy products, and the greatest diversity was found in hard and semi-hard cheeses. Furthermore, Decontardi et al. [38] reported the presence of fungi in hard cheese and detected the presence of the mycotoxin ochratoxin A, indicating that the identified species was a mycotoxin producer. Considering that it is common to isolate fungi from the production environment, management strategies that prevent environmental contamination in dairy industries are essential for maintaining product quality and food safety, since many of these fungi can represent a source of contamination and can generate economic losses for the industry [10].

Regarding production environments, locations where a greater variety of fungal genera were detected included the production room and the cheese manufacturing tank, which reinforces the importance of applying quality management systems, such as GMP (Good Manufacturing Practice) and HACCP (Hazard Analysis Critical Control Point), to minimize problems associated with microbiological contamination.

In the present study, we identified 85 species and distributed in 16 genera belonging to the Ascomycota phylum, which are the most frequently described in dairy products [5,39]. Among all the genera identified, only one does not belong to the Ascomycota phylum (*Riopa* = Basidiomycota). The genera most commonly and frequently found in dairy products are *Penicillium* and *Cladosporium*, of the Eurotiomycetes class [13,16,17,40], which were also the main genera identified in this study. One of the biggest problems of these genera in cheeses is that they can degrade compounds such as sorbic acid, potassium sorbate, and 1,3 pentadiene, which causes a strong off-flavor and unpleasant odor, called “kerosene odor” [10]. In addition, the visible growth of an undesirable fungus on any type of cheese results in immediate consumer dissatisfaction. 

The predominant presence of *Penicillium* in several types of semi-hard and hard cheeses can be attributed to an adaptive response to water stress, mainly because these cheeses generally have water activity below the optimal level required for fungal growth [41]. On the other hand, the prevalence of *Cladosporium* in the dairy production environment, rather than in dairy products, is largely due to the species of this genus generally being slow growing and commonly disseminated through air. They can also be psychrotolerant and xerotolerant [5].

Another genus largely reported and associated with dairy product contamination is *Aspergillus* [40,42,43]. However, despite being widely reported in the literature, this genus was not identified in the spoiled products, only from the production environment. On the other hand, the genera *Phaeosphaeria* and *Fusarium* were identified as contaminants in the dairy products, but they were not identified in the dairy processing environment, most likely due to the great diversity of genera found. One of the major problems associated with the occurrence of filamentous fungi in food products is mycotoxin contamination. Mycotoxins are toxic secondary metabolites produced mainly by the species in the genera of *Aspergillus*, *Penicillium*, and *Fusarium*, among others [13], all of which were identified in the current study.

As these genera were identified at great frequency in this study, the possible problems associated with mycotoxin contamination must be considered. The most dangerous mycotoxins reported thus far in cheese are Ochratoxin A and Aflatoxin M1 (AFM1), and are produced by fungi through direct or indirect contamination of milk (such as contamination of animal feed), respectively. This has been well reviewed by Hymery et al. [44]. Akinyemi et al. [45] reported the presence of several mycotoxins in three types of milk (camel, cow, and goat milk) and detected aflatoxins, alternariol, monomethyl ether, citrinin, dihydrocitrinone, enniatins, ochratoxin, and sterigmatocystin. Fontaine et al. [11] detected the presence of mycotoxins in cheeses, such as roquefortine C and mycophenolic acid; however, they did not detect the presence of AFM1. Although mycotoxin management in cheese is important, identifying and detecting mycotoxigenic fungal strains is fundamental; in order to track the possible origins of metabolite production. A study carried out by Anelli et al. [13] detected mycotoxigenic species of *Aspergillus* and *Penicillium* isolated from cheese and was able to correlate these species with the production of mycotoxins in cheese rinds during ripening.

The main effects of mycotoxins in the human body are liver and kidney toxicity, immune system destabilization, fetal toxicity, and carcinogenicity; however, it is not easy to prove that fungal species can produce mycotoxin or whether the mycotoxin will cause any damage if detected in a product. Nevertheless, recent models and some epidemiological data are enough to conclude that mycotoxins pose a danger, and that more studies on the risk of exposure to toxins should be further studied and validated [46,47]. 

Other genera that were identified in this study have also been reported by other authors as contaminants of dairy products and production environments, such as *Didymella* and *Fusarium*, which were identified by Cenci-Goga et al. [48] at low prevalence (1.5% each one). Garnier et al. [17] also identified the genera mentioned above and found *Phaeosphaeria* at a lower frequency (1 isolated), as in the present study. In addition, *Trichoderma* has been described in dairy production environments and products [15] and was isolated from the gorgonzola ripening room in the current work. The genus *Paecilomyces* was also described [16]. In fact, the diversity of genera and some species of filamentous fungi in dairy products and plants is well recognized. However, it is known that the biodiversity of a given region can favor the prevalence of one type of fungi over others. As an example, some studies report the prevalence of *Penicillium* in certain regions, such as in Spanish, French, and Italian dairy products [17,49,50]. Marín et al. [41] reported that *Geotrichum* and *Fusarium* were the genera most frequently isolated from Spanish milk samples. Other studies consider *Aspergillus*, *Cladosporium*, *Mucor*, and *Peniciliium* as the major contaminants in Egytian dairy products [42,51]. Nevertheless, the genera identified in this work, such as *Riopa*, *Hypoxylon*, *Montagnula*, *Clonostachys*, *Rhinocladiella*, *Coniochaeta*, and *Bipolaris*, are not commonly found in dairy production environments. In this present work, *Riopa*, *Hypoxylon*, *Montagnula*, *Clonostachys*, and *Coniochaeta* have been identified for the first time from a dairy production environment. *Bipolaris* and *Rhinocladiella* genera were also identified in a study conducted by Mbareche et al. [52] and by Moubasher et al. [53] from a dairy farm and Roquefort cheese, respectively. The latter was found over four decades ago and has not been reported further.

*Bipolaris* is a genus of dark conidia fungi in the phylum Ascomycota and is generally reported as a plant and animal pathogen [54]. The genus *Riopa*, belonging to the Basidiomycota phylum, contains two species, *Riopa metamorphosa* and *Riopa pudens*. This genus has already been classified as a synonym of another genus *Ceriporia* because they are extremely similar phenotypically and genotypically [55]. They are a common soil borne fungi and are decomposers of tree wood and can cause white rot [56]. This may be the first report of this genus being associated with dairy environments, and despite not being reported in food, the current study frequently isolated this genus from various types of dairy production environments.

*H. griseobrunneum* was reported in our results, and it is an atypical fungus isolated from dairy products and the dairy environment, probably due to it being an endophytic filamentous fungus. The morphological characteristics of this species are well known, and the brown color on the surface of the colony facilitates its identification at the species level [32]. In addition, *Montagnula* was also described in our results. *Montagnula* is a genus that in the last 10 years has undergone several changes in its taxonomy and with many new species inclusions [57]. Perhaps this is the reason for not having a greater knowledge of the genus being associated with dairy products and environments, considering that the inclusion of the new species into molecular datasets is recent. This genus is associated with growth on dead wood, branches, stems, bark, and leaves [57] and is therefore a contaminant present in the dairy industries.

*Clonostachys* spp. are commonly found in temperate climates, typically on decomposing plant material; however, it is a non-pathogenic genus that may even have biocontrol characteristics for phytopathogens [58,59]. *C. rosae* was recently described by Wanasinghe et al. [34], but it was not associated with dairy and food environments. *R. similis*, another species identified in this study, was described by De Hoog et al. [60] and originally isolated from a chronic cutaneous ulcer of a patient in the state of Minas Gerais, Brazil. It is considered a pathogenic fungus, and its colonies present characteristics of a medium with a black, dry, and velvety center; the budding cells are abundant, with the development of germ cells and brown hyphae. The morphological characteristics described coincide with our study (Figure 3) and can be compared with other studies [33,61]. Although *R. similis* is a recently described species, several studies are being carried out in America and Europe to better understand this filamentous fungus and its pathogenicity, mainly in the medical field [33,61,62]. However, this is the first report of *R. similis* associated with food or the food environment. We consider this fact important to carry out further studies in this area, as the discovery of new species and genera with no previous association with the food industry may have negative consequences. 

In recent years, the taxonomy of fungi has increasingly changed, and many species are being reclassified [17]. New phylogenetic species are recognized as “species complexes” because they have few morphological differences, often making identification to the species level or even to genera difficult. In this way, our phylogeny shows that the isolates that were not identified display great similarity between them, such as, the representatives from Didymellaceae, which include *Epicoccum* and *Didymella*. These genera are extremely similar even in their morphological characteristics [63]. The inclusion of several species into these families and a new DNA marker (*rpb2*) for better identification at the species level were suggested [63]. It is important to use genetic markers for several species-level identifications. However, for an initial and general tracking of the presence of fungi in certain environments, it is important to begin from a starting point, generally the ITS region. Now, for specific identification to the species level and/or specific genera, the use of multiple or different DNA markers is essential.

Finally, considering the importance of filamentous fungi diversity present in dairy processing plants, it is important to emphasize that, clean/sterile areas must be designated and maintained, to avoid possible fungal contamination of the entire production environment and dairy products. With the present study, it was possible to find a biodiversity of filamentous fungi in dairy products and environments, and some strains were found for the first time in the dairy environment.

In this way, understanding the specific biodiversity of fungi from each region and stage of the production system is very important, in order to develop new strategies to control spoilage. Furthermore, more studies are required to evaluate new fungi species identified within the production environment, to better understand their role.

## Figures and Tables

**Table 1:** Diversity and number of fungi isolated from spoiled dairy products (*n* = number of samples).

Identification and Number of Isolates	Mozzarela Cheese (*n* = 3)	Reino Cheese (*n* = 2)	Montanhês Cheese (*n* = 3)	Parmesan Cheese (*n* = 2)	Ghee Butter (*n* = 2)	Gorgonzola Cheese (*n* = 1)	Provolone Cheese (*n* = 2)	Processed Cheese (*n* = 2)	Coalho Cheese (*n* = 1)	Total % in Spoiled Dairy Products	Total % in Air, Equipment, and Tools
*Penicillium* (36)	2	5	2	4	2	5	3	-	2	25 (22.94%)	11 (10.09%)
*Cladosporium* (23)	-	-	2	-	-	-	-	2	2	6 (5.50%)	17 (15.60%)
*Nigrospora* (5)	-	-	-	-	-	-	-	-	-	-	5 (4.58%)
*Riopa* (5)	-	-	-	-	-	-	-	-	-	-	5 (4.58%)
*Aspergillus* (4)	-	-	-	-	-	-	-	-	-	-	4 (3.67%)
*Hipoxylon* (2)	-	-	-	-	-	-	-	-	-	-	2 (1.83%)
*Fusarium* (1)	-	-	1	-	-	-	-	-	-	1 (0.92%)	-
*Montagnula* (1)	-	-	-	-	-	-	-	-	-	-	1 (0.92%)
*Clonostachys* (1)	-	-	-	-	-	-	-	-	-	-	1(0.92%)
*Phaeosphaeria* (1)	-	-	-	-	-	-	-	-	1	1(0.92%)	-
*Rhinocladiella* (1)	-	-	-	-	-	-	-	-	-	-	1(0.92%)
*Coniochaeta* (1)	-	-	-	-	-	-	-	-	-	-	1(0.92%)
*Trichoderma* (1)	-	-	-	-	-	-	-	-	-	-	1(0.92%)
*Paecilomyces* (1)	-	-	-	-	-	-	-	-	-	-	1(0.92%)
*Didymella* (1)	-	-	-	-	-	-	-	-	-	-	1(0.92%)
*Bipolaris* (1)	-	-	-	-	-	-	-	-	-	-	1(0.92%)
Not identified (24)	3	2	2	-	-	-	-	1	-	8 (7.33%)	16(14.68%)
Total	5	7	7	4	5	3	3	3	5	41	68
Total (%)	12.19	17.07	17.07	9.76	12.19	7.33	7.33	7.33	12.19	37.61	62.39

(-) not detected.

**Table 2:** Diversity of spoilage fungi isolated from dairy environments (factory air, equipment and tools).

Identification	Ware House	Cold Chamber	Ghee Stock Room	Product Refrigerators	Cheese Ripening Room	Gorgonzola Ripening Room	Production Room	Cheese Packing Room	Brine Room	Production Tables	Cheese Containers	Cheese Packaging	Cheese Tank
*Penicillium* sp.	-	1	-	-	1	-	-	1	-	-	6	-	2
*Cladosporium* sp.	1	1	2	2	4	-	2	1	1	1	-	1	1
*Nigrospora* sp.	-	-	1	-	-	-	1	2	-	-	-	-	1
*Riopa* sp.	-	1	-	1	1	-	1	-	-	-	-	-	1
*Aspergillus* sp.	1	-	-	1	-	-	1	-	-	-	-	-	1
*Hipoxylon* sp.	-	-	-	-	-	-	-	-	-	-	-	-	2
*Fusarium* sp.	-	-	-	-	-	-	-	-	-	-	-	-	-
*Montagnula* sp.	-	-	-	-	-	-	1	-	-	-	-	-	-
*Clonostachys* sp.	-	-	1	-	-	-	-	-	-	-	-	-	-
*Phaeosphaeria* sp.	-	-	-	-	-	-	-	-	-	-	-	-	-
*Rhinocladiella* sp.	-	-	-	-	-	-	1	-	-	-	-	-	-
*Coniochaeta* sp.	-	-	-	-	-	-	1	-	-	-	-	-	-
*Trichoderma* sp.	-	-	-	-	-	1	-	-	-	-	-	-	-
*Paecilomyces* sp.	-	-	-	-	-	-	-	-	1	-	-	-	-
*Didymella* sp.	-	-	-	-	-	-	-	-	-	-	-	1	-
*Bipolaris* sp.	-	-	-	-	-	-	-	-	-	-	1	-	-
Not identified	3	-	2	-	1	-	6	1	-	1	1	1	-
Total	5	3	6	4	7	1	14	5	2	2	8	3	8
Total (%)	7.35	4.42	8.82	5.89	10.29	1.47	20.59	7.35	2.94	2.94	11.76	4.42	11.76

(-) not detected.

**Table 3:** Species identified according to BLAST of the NCBI database.

Sample	Length (nt)	Identification		BLASTN	
			Query Coverage	Percent Identify	Accession Number
1.21	570	*H. griseobrunneum*	97%	98.92%	NR_155184.1
4.24	640	*R. similis*	96%	100.00%	NR_166008.1
4.27	600	*C. rosae*	94%	95.13%	NR_157509.1
4.38	630	*P. maximus*	90%	96.84%	NR_149329.1

## Data Availability

Data is contained within the article or supplementary material.

## Supplementary Materials

The following supporting information can be downloaded at: https://www.mdpi.com/article/10.3390/foods12010153/s1. Table S1: Identified fungal isolates based on BLASTN search on the RefSeq curated dataset for Internal transcribed spacer region (ITS) from Fungi and reference material, on NCBI; Figure S1: The evolutionary history, using ITS region nucleotide sequences, was inferred using the Maximum Likelihood method and Kimura 2-parameter model [28]. The tree with the highest log likelihood is shown. A discrete Gamma distribution was used to model evolutionary rate differences among sites. All positions with less than 95% site coverage were eliminated (partial deletion option). There was a total of 438 positions in the final dataset. Numbers at branches indicate percentages of bootstrap values (>20%) obtained from 1000 replicates. *Entorrhiza citriformis* and *E. parvula* were used as outgroup.

## References

[1] Food and Agriculture Organization of the United Nations The State of Food and Agriculture (2019). Moving Forward on Food Loss and Waste Reduction.

[2] United Nations Environment Programme UNEP Food Waste Index—Report 2021. https://www.unep.org/resources/report/unep-food-waste-index-report-2021.

[3] United States Department of Agriculture (USDA), Environmental Protection Agency (EPA) U.S. Food Loss & Waste 2030 Champions. Milestones Report—May 2021. https://www.usda.gov/sites/default/files/documents/food-loss-waste-champions-report.pdf.

[4] Brooks J.C., Martinez B., Stratton J., Bianchini A., Krokstrom R., Hutkins R. (2012). Survey of Raw Milk Cheeses for Microbiological Quality and Prevalence of Foodborne Pathogens. Food Microbiol..

[5] Pitt John I., Hocking A.D. (2009). The Ecology of Fungal Food Spoilage. Fungi and Food Spoilage.

[6] Pattono D., Grosso A., Stocco P.P., Pazzi M., Zeppa G. (2013). Survey of the Presence of Patulin and Ochratoxin A in Traditional Semi-Hard Cheeses. Food Control.

[7] Ráduly Z., Szabó L., Madar A., Pócsi I., Csernoch L. (2020). Toxicological and Medical Aspects of *Aspergillus*-Derived Mycotoxins Entering the Feed and Food Chain. Front. Microbiol..

[8] Parussolo G., Bernardi A.O., Garcia M.V., Stefanello A., dos Santos Silva T., Copetti M.V. (2019). Fungi in Air, Raw Materials and Surface of Dry Fermented Sausage Produced in Brazil. LWT.

[9] Pinches S.E., Apps P. (2007). Production in Food of 1,3-Pentadiene and Styrene by *Trichoderma* Species. Int. J. Food Microbiol..

[10] Leyva Salas M., Mounier J., Valence F., Coton M., Thierry A., Coton E. (2017). Antifungal Microbial Agents for Food Biopreservation—A Review. Microorganisms.

[11] Fontaine K., Passeró E., Vallone L., Hymery N., Coton M., Jany J.-L., Mounier J., Coton E. (2015). Occurrence of Roquefortine C, Mycophenolic Acid and Aflatoxin M1 Mycotoxins in Blue-Veined Cheeses. Food Control.

[12] Dobson A.D.W., McSweeney P.L.H., Fox P.F., Cotter P.D., Everett D.W. (2017). Chapter 23—Mycotoxins in Cheese. Cheese.

[13] Anelli P., Haidukowski M., Epifani F., Cimmarusti M.T., Moretti A., Logrieco A., Susca A. (2019). Fungal Mycobiota and Mycotoxin Risk for Traditional Artisan Italian Cave Cheese. Food Microbiol.

[14] Instituto Brasileiro de Geografia e Estatística (IBGE) Pesquisa Trimestral Do Leite. https://www.ibge.gov.br/estatisticas/economicas/agricultura-e-pecuaria/9209-pesquisa-trimestral-do-leite.html?=&t=destaques.

[15] Kure C.F., Wasteson Y., Brendehaug J., Skaar I. (2001). Mould Contaminants on Jarlsberg and Norvegia Cheese Blocks from Four Factories. Int. J. Food Microbiol..

[16] Kure C.F., Skaar I., Brendehaug J. (2004). Mould Contamination in Production of Semi-Hard Cheese. Int. J. Food Microbiol..

[17] Garnier L., Valence F., Pawtowski A., Auhustsinava-Galerne L., Frotté N., Baroncelli R., Deniel F., Coton E., Mounier J. (2017). Diversity of Spoilage Fungi Associated with Various French Dairy Products. Int. J. Food Microbiol..

[18] Buehler A.J., Evanowski R.L., Wiedmann M., Martin N.H. (2019). Internal Transcribed Spacer (ITS) Sequence-Based Characterization of Fungal Isolates from Multiple Yogurt Facilities—A Case Study. J. Dairy Sci..

[19] Snyder A.B., Churey J.J., Worobo R.W. (2019). Association of Fungal Genera from Spoiled Processed Foods with Physicochemical Food Properties and Processing Conditions. Food Microbiol..

[20] de Souza T.P., Evangelista S.R., Passamani F.R.F., Bertechini R., de Abreu L.R., Batista L.R. (2021). Mycobiota of Minas Artisanal Cheese: Safety and Quality. Int. Dairy J..

[21] le Lay C., Mounier J., Vasseur V., Weill A., le Blay G., Barbier G., Coton E. (2016). In Vitro and in Situ Screening of Lactic Acid Bacteria and Propionibacteria Antifungal Activities against Bakery Product Spoilage Molds. Food Control.

[22] Legnani P., Leoni E., Berveglieri M., Mirolo G., Alvaro N. (2004). Hygienic Control of Mass Catering Establishments, Microbiological Monitoring of Food and Equipment. Food Control.

[23] Visagie C.M., Hirooka Y., Tanney J.B., Whitfield E., Mwange K., Meijer M., Amend A.S., Seifert K.A., Samson R.A. (2014). *Aspergillus, Penicillium* and *Talaromyces* Isolated from House Dust Samples Collected around the World. Stud. Mycol..

[24] White T.J., Bruns T.D., Lee S., Taylor J.W., Innis M.A., Gelfand D.H., Sninsky J., White T.J. (1990). Amplification and Direct Sequencing of Fungal Ribosomal RNA Genes for Phylogenetics. PCR Protocols: A Guide to Methods and Applications.

[25] Tamura K., Stecher G., Kumar S. (2021). MEGA11: Molecular Evolutionary Genetics Analysis Version 11. Mol. Biol. Evol..

[26] Edgar R.C. (2004). MUSCLE: Multiple Sequence Alignment with High Accuracy and High Throughput. Nucleic Acids Res..

[27] Felsenstein J. (1981). Journal of Molecular Evolution Evolutionary Trees from DNA Sequences: A Maximum Likelihood Approach. J Mol. Evol..

[28] Kimura M. (1980). A Simple Method for Estimating Evolutionary Rates of Base Substitutions Through Comparative Studies of Nucleotide Sequences. J. Mol. Evol..

[29] Tamura K. (1992). Estimation of the Number of Nucleotide Substitutions When There Are Strong Transition-Transversion and G+C-Content Biases. Mol. Biol. Evol..

[30] Felsenstein J. (1985). Confidence Limits on Phylogenies: An Approach Using the Bootstrap. Evolution.

[31] Letunic I., Bork P. (2021). Interactive Tree Of Life (ITOL) v5: An Online Tool for Phylogenetic Tree Display and Annotation. Nucleic Acids Res..

[32] Kuhnert E., Fournier J., Peršoh D., Luangsa-ard J.J.D., Stadler M. (2014). New *Hypoxylon* Species from Martinique and New Evidence on the Molecular Phylogeny of *Hypoxylon* Based on ITS RDNA and β-Tubulin Data. Fungal Divers..

[33] Abdolrasouli A., Gibani M.M., de Groot T., Borman A.M., Hoffman P., Azadian B.S., Mughal N., Moore L.S.P., Johnson E.M., Meis J.F. (2021). A Pseudo-Outbreak of *Rhinocladiella Similis* in a Bronchoscopy Unit of a Tertiary Care Teaching Hospital in London, United Kingdom. Mycoses.

[34] Wanasinghe D.N., Phukhamsakda C., Hyde K.D., Jeewon R., Lee H.B., Gareth Jones E.B., Tibpromma S., Tennakoon D.S., Dissanayake A.J., Jayasiri S.C. (2018). Fungal Diversity Notes 709–839: Taxonomic and Phylogenetic Contributions to Fungal Taxa with an Emphasis on Fungi on *Rosaceae*. Fungal Divers..

[35] Zhang Y.-J., Herrera-Balandrano D.D., Shi X.-C., Wang S.-Y., Laborda P. (2022). Biocontrol of *Colletotrichum Brevisporum* in Soybean Using a New Genistein-Producing *Paecilomyces* Strain. Biol. Control.

[36] Codex Alimentarius Commission General Standar for Cheese (CXS 283-1978). https://www.fao.org/fao-who-codexalimentarius/sh-proxy/en/?lnk=1&url=https%253A%252F%252Fworkspace.fao.org%252Fsites%252Fcodex%252FStandards%252FCXS%2B283-1978%252FCXS_283e.pdf.

[37] Nájera A.I., Nieto S., Barron L.J.R., Albisu M. (2021). A Review of the Preservation of Hard and Semi-Hard Cheeses: Quality and Safety. Int. J. Environ. Res. Public Health.

[38] Decontardi S., Mauro A., Lima N., Battilani P. (2017). Survey of Penicillia Associated with Italian Grana Cheese. Int. J. Food Microbiol..

[39] Lund F., Filtenborg O., Frisvad J.C. (1995). Associated Mycoflora of Cheese. Food Microbiol..

[40] Kandasamy S., Park W.S., Yoo J., Yun J., Kang H.B., Seol K.-H., Oh M.-H., Ham J.S. (2020). Characterisation of Fungal Contamination Sources for Use in Quality Management of Cheese Production Farms in Korea. Asian-Australas. J. Anim. Sci..

[41] Marín P., Palmero D., Jurado M. (2015). Occurrence of Moulds Associated with Ovine Raw Milk and Cheeses of the Spanish Region of Castilla La Mancha. Int. J. Dairy Technol..

[42] Moubasher A.-A.H., Abdel-Sater M.A., Soliman Z.S.M. (2018). Yeasts and Filamentous Fungi Associated with Some Dairy Products in Egypt. J. Mycol. Med..

[43] Torkar K.G., Vengušt A. (2008). The Presence of Yeasts, Moulds and Aflatoxin M1 in Raw Milk and Cheese in Slovenia. Food Control.

[44] Hymery N., Vasseur V., Coton M., Mounier J., Jany J.L., Barbier G., Coton E. (2014). Filamentous Fungi and Mycotoxins in Cheese: A Review. Compr. Rev. Food Sci. Food Saf..

[45] Akinyemi M.O., Braun D., Windisch P., Warth B., Ezekiel C.N. (2022). Assessment of Multiple Mycotoxins in Raw Milk of Three Different Animal Species in Nigeria. Food Control.

[46] Pinhão M., Tavares A.M., Loureiro S., Louro H., Alvito P., Silva M.J. (2020). Combined Cytotoxic and Genotoxic Effects of Ochratoxin A and Fumonisin B1 in Human Kidney and Liver Cell Models. Toxicol Vitr..

[47] Claeys L., de Saeger S., Scelo G., Biessy C., Casagrande C., Nicolas G., Korenjak M., Fervers B., Heath A.K., Krogh V. (2022). Mycotoxin Exposure and Renal Cell Carcinoma Risk: An Association Study in the EPIC European Cohort. Nutrients.

[48] Cenci-Goga B., Cruciani D., Crotti S., Karama M., Ylldlrlm G., Bulut M., Marino C., Grispoldi L. (2021). Diversity of Yeasts and Moulds in Dairy Products from Umbria, Central Italy. J. Dairy Res..

[49] Barrios M.J., Medina L.M., Lopez M.C., Jordano R. (1998). Fungal Biota Isolated from Spanish Cheeses. J. Food Saf..

[50] Montagna M.T., Santacroce M.P., Spilotros G., Napoli C., Minervini F., Papa A., Dragoni I. (2004). Investigation of Fungal Contamination in Sheep and Goat Cheeses in Southern Italy. Mycopathologia.

[51] Seddek N.H., Gomah N.H., Osman D.M. (2016). Fungal Flora Contaminating Egyptian Ras Cheese with Reference to Their Toxins and Enzymes. Food Sci. Technol..

[52] Mbareche H., Veillette M., Bilodeau G.J., Duchaine C. (2019). Fungal Aerosols at Dairy Farms Using Molecular and Culture Techniques. Sci. Total Environ..

[53] Moubasher A.H., Abdel-Kader M.I.A., El-Kady I.A. (1979). Toxigenic Fungi Isolated from Roquefort Cheese. Mycopathologia.

[54] Giri D.K., Sims W.P., Sura R., Cooper J.J., Gavrilov B.K., Mansell J. (2011). Cerebral and Renal Phaeohyphomycosis in a Dog Infected with *Bipolaris* Species. Vet. Pathol..

[55] Miettinen O., Spirin V., Vlasák J., Rivoire B., Stenroos S., Hibbett D.S. (2016). Polypores and Genus Concepts in Phanerochaetaceae (*Polyporales*, Basidiomycota). MycoKeys.

[56] Justo A., Miettinen O., Floudas D., Ortiz-Santana B., Sjökvist E., Lindner D., Nakasone K., Niemelä T., Larsson K.-H., Ryvarden L. (2017). A Revised Family-Level Classification of the *Polyporales* (*Basidiomycota*). Fungal Biol..

[57] Mapook A., Hyde K.D., McKenzie E.H.C., Jones E.B.G., Bhat D.J., Jeewon R., Stadler M., Samarakoon M.C., Malaithong M., Tanunchai B. (2020). Taxonomic and Phylogenetic Contributions to Fungi Associated with the Invasive Weed *Chromolaena Odorata* (Siam Weed). Fungal Divers..

[58] Schroers H.-J. (2001). A Monograph of Bionectria (Ascomycota, Hypocreales, Bionectriaceae) and Its Clonostachys Anamorphs. Stud. Mycol..

[59] Moraga-Suazo P., Opazo A., Zaldúa S., González G., Sanfuentes E. (2011). Evaluation of *Trichoderma* Spp. and *Clonostachys* Spp. Strains to Control *Fusarium Circinatum* in *Pinus Radiata* Seedlings. Chil. J. Agric. Res..

[60] De Hoog G.S., Vicente V., Caligiorne R.B., Kantarcioglu S., Tintelnot K., van den Ende A.H.G.G., Haase G. (2003). Species Diversity and Polymorphism in the *Exophiala Spinifera* Clade Containing Opportunistic Black Yeast-Like Fungi. J. Clin. Microbiol..

[61] Heidrich D., González G.M., Pagani D.M., Ramírez-Castrillón M., Scroferneker M.L. (2017). Chromoblastomycosis Caused by *Rhinocladiella Similis*: Case Report. Med. Mycol. Case Rep..

[62] De Andrade T.S., de Almeida A.M.Z., Basano S.d.A., Takagi E.H., Szeszs M.W., Melhem M.S.C., Albuquerque M., Camargo J.d.S.A.A., Gambale W., Camargo L.M.A. (2020). Chromoblastomycosis in the Amazon Region, Brazil, Caused by *Fonsecaea Pedrosoi, Fonsecaea Nubica*, and *Rhinocladiella Similis*: Clinicopathology, Susceptibility, and Molecular Identification. Med. Mycol..

[63] Hou L.W., Groenewald J.Z., Pfenning L.H., Yarden O., Crous P.W., Cai L. (2020). The Phoma-like Dilemma. Stud. Mycol..

