# Supplementary material for: Diversity of Filamentous Fungi Associated with Dairy Processing Environments and Spoiled Products in Brazil
Source: Foods. 2022 Dec 28;12(1):153. doi: 10.3390/foods12010153 (PMC9818152; doi:10.3390/foods12010153)
Supplement: Supplementary file 1 [file foods-12-00153-s001.zip › foods-2065744-supplementary.pdf]

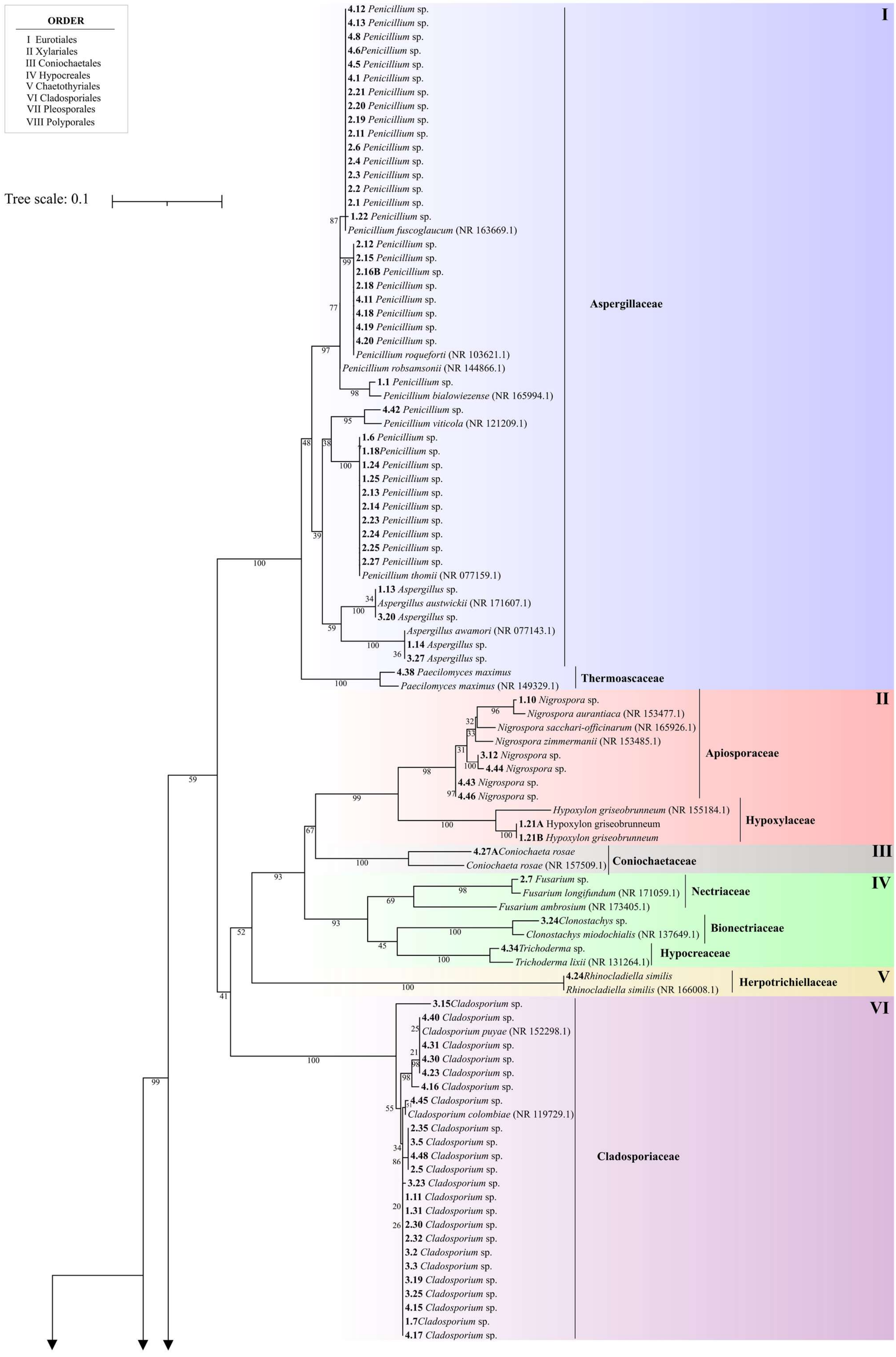
ORDER
I Eurotiales
II Xylariales
III Coniochaetales
IV Hypocreales
V Chaetothyriales
VI Cladosporiales
VII Pleosporales
VIII Polyporales
Tree scale: 0.1
4.12 Penicillium sp.
4.13 Penicillium sp.
4.8 Penicillium sp.
4.6Penicillium sp.
4.5 Penicillium sp.
4.1 Penicillium sp.
2.21 Penicillium sp.
2.20 Penicillium sp.
2.19 Penicillium sp.
2.11 Penicillium sp.
2.6 Penicillium sp.
2.4 Penicillium sp.
2.3 Penicillium sp.
2.2 Penicillium sp.
2.1 Penicillium sp.
1.22 Penicillium sp.
Penicillium fuscoglaucum (NR 163669.1)
2.12 Penicillium sp.
2.15 Penicillium sp.
2.16B Penicillium sp.
2.18 Penicillium sp.
4.11 Penicillium sp.
4.18 Penicillium sp.
4.19 Penicillium sp.
4.20 Penicillium sp.
Penicillium roqueforti (NR 103621.1)
Penicillium robsamsonii (NR 144866.1)
1.1 Penicillium sp.
Penicillium bialowiezense (NR 165994.1)
4.42 Penicillium sp.
Penicillium viticola (NR 121209.1)
1.6 Penicillium sp.
1.18Penicillium sp.
1.24 Penicillium sp.
1.25 Penicillium sp.
2.13 Penicillium sp.
2.14 Penicillium sp.
2.23 Penicillium sp.
2.24 Penicillium sp.
2.25 Penicillium sp.
2.27 Penicillium sp.
Penicillium thomii (NR 077159.1)
1.13 Aspergillus sp.
Aspergillus austwickii (NR 171607.1)
3.20 Aspergillus sp.
Aspergillus awamori (NR 077143.1)
1.14 Aspergillus sp.
3.27 Aspergillus sp.
4.38 Paecilomyces maximus
Paecilomyces maximus (NR 149329.1)
I
Aspergillaceae
Thermoascaceae
II
1.10 Nigrospora sp.
Nigrospora aurantiaca (NR 153477.1)
Nigrospora sacchari-officinarum (NR 165926.1)
Nigrospora zimmermanii (NR 153485.1)
3.12 Nigrospora sp.
4.44 Nigrospora sp.
4.43 Nigrospora sp.
4.46 Nigrospora sp.
Apiosporaceae
Hypoxylon griseobrunneum (NR 155184.1)
1.21A Hypoxylon griseobrunneum
1.21B Hypoxylon griseobrunneum
Hypoxylaceae
III
4.27AConiochaeta rosae
Coniochaeta rosae (NR 157509.1)
Coniochaetaceae
IV
2.7 Fusarium sp.
Fusarium longifundum (NR 171059.1)
Fusarium ambrosium (NR 173405.1)
Nectriaceae
3.24Clonostachys sp.
Clonostachys miodochialis (NR 137649.1)
Bionectriaceae
4.34Trichoderma sp.
Trichoderma lixii (NR 131264.1)
Hypocreaceae
V
4.24Rhinocladiella similis
Rhinocladiella similis (NR 166008.1)
Herpotrichiellaceae
VI
3.15Cladosporium sp.
4.40 Cladosporium sp.
Cladosporium puyae (NR 152298.1)
4.31 Cladosporium sp.
4.30 Cladosporium sp.
4.23 Cladosporium sp.
4.16 Cladosporium sp.
4.45 Cladosporium sp.
Cladosporium colombiae (NR 119729.1)
2.35 Cladosporium sp.
3.5 Cladosporium sp.
4.48 Cladosporium sp.
2.5 Cladosporium sp.
3.23 Cladosporium sp.
1.11 Cladosporium sp.
1.31 Cladosporium sp.
2.30 Cladosporium sp.
2.32 Cladosporium sp.
3.2 Cladosporium sp.
3.3 Cladosporium sp.
3.19 Cladosporium sp.
3.25 Cladosporium sp.
4.15 Cladosporium sp.
1.7Cladosporium sp.
4.17 Cladosporium sp.
Cladosporiaceae

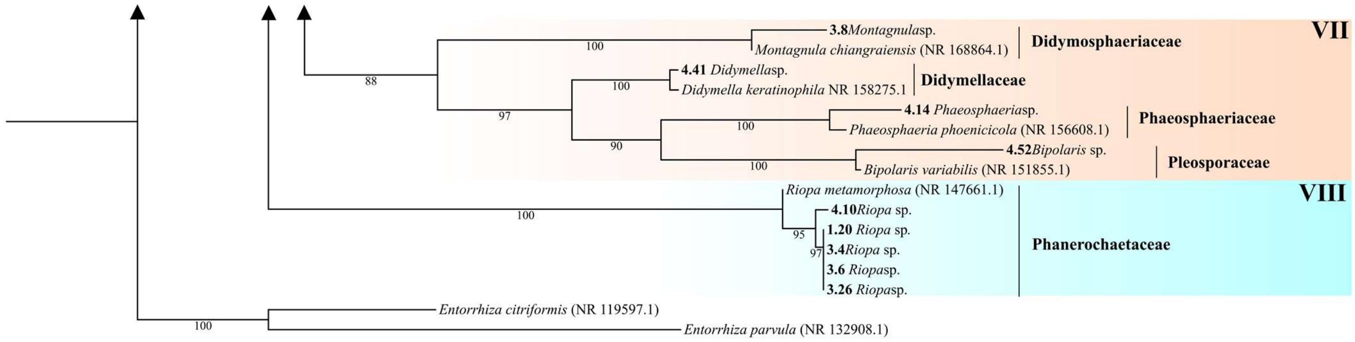

Supplementary Figure S1. The evolutionary history, using ITS region nucleotide sequences, was inferred using the Maximum Likelihood method and Kimura 2-parameter model [28]. The tree with the highest log likelihood is shown. A discrete Gamma distribution was used to model evolutionary rate differences among sites. All positions with less than 95% site coverage were eliminated (partial deletion option). There was a total of 438 positions in the final dataset. Numbers at branches indicate percentages of bootstrap values (>20%) obtained from 1,000 replicates. *Entorrhiza citriformis* and *E. parvula* were used as outgroup.

Supplementary Table S1. Identified fungal isolates based on BLASTN search on the RefSeq curated dataset for Internal transcribed spacer region (ITS) from Fungi and reference material, on NCBI.

Sample code	Length (nt)	Identification	BLASTN			
			Scientific name	Accession number	Query coverage	Percent identity
4.26	569	Not identified	*Epicoccum phragmospora*	NR_165920.1	98%	97.68%
			Didymella keratinophila	NR_158275.1	98%	96.97%
			Ascochyta phacae	NR_135942.1	100%	96.49%
4.47	569	Not identified	*Epicoccum phragmospora*	NR_165920.1	98%	97.68%
			Ascochyta phacae	NR_135942.1	100%	96.84%
			Didymella keratinophila	NR_158275.1	98%	96.97%
3.16	569	Not identified	*Epicoccum phragmospora*	NR_165920.1	98%	97.86%
			Ascochyta phacae	NR_135942.1	100%	97.36%
			Didymella keratinophila	NR_158275.1	98%	97.15%
3.7	569	Not identified	*Epicoccum phragmospora*	NR_165920.1	98%	97.68%
			Ascochyta phacae	NR_135942.1	100%	96.84%
			Didymella keratinophila	NR_158275.1	98%	96.61%
1.3	569	Not identified	*Epicoccum phragmospora*	NR_165920.1	98%	97.68%
			Didymella keratinophila	NR_158275.1	98%	96.97%
			Ascochyta phacae	NR_135942.1	100%	96.49%
1.12	569	Not identified	*Epicoccum phragmospora*	NR_165920.1	98%	97.68%
			Didymella keratinophila	NR_158275.1	98%	96.97%
			Ascochyta phacae	NR_135942.1	100%	96.49%
2.9	569	Not identified	*Epicoccum phragmospora*	NR_165920.1	98%	97.68%
			Didymella keratinophila	NR_158275.1	98%	96.97%
			Ascochyta phacae	NR_135942.1	100%	96.49%
2.26	569	Not identified	*Epicoccum phragmospora*	NR_165920.1	98%	97.68%
			Didymella keratinophila	NR_158275.1	98%	96.97%
			Ascochyta phacae	NR_135942.1	100%	96.49%
2.36	569	Not identified	*Epicoccum phragmospora*	NR_165920.1	98%	97.68%
			Didymella keratinophila	NR_158275.1	98%	96.97%
			Ascochyta phacae	NR_135942.1	100%	96.49%
3.1	569	Not identified	*Epicoccum phragmospora*	NR_165920.1	98%	97.68%
			Didymella keratinophila	NR_158275.1	98%	96.97%
			Ascochyta phacae	NR_135942.1	100%	96.49%

3.13	566	Not identified	*Stagonosporopsis lupini*	NR_160205.1	95%	98.89%
			Stagonosporopsis valerianellae	NR_160109.1	96%	97.80%
			Allophoma cylindrispora	NR_158276.1	96%	97.63%
4.22	558	Not identified	*Didymella keratinophila*	NR_158275.1	98%	97.13%
			Epicoccum thailandicum	NR_152926.1	91%	99.41%
			Epicoccum phragmospora	NR_165920.1	98%	96.60%
4.49	567	Not identified	*Didymella keratinophila*	NR_158275.1	98%	98.75%
			Epicoccum phragmospora	NR_165920.1	98%	97.50%
			Ascochyta phacae	NR_135942.1	100%	97.00%
4.7	602	Not identified	-	-	-	-
1.16	634	Not identified	-	-	-	-
4.28	617	Not identified	-	-	-	-
3.22	612	Not identified	-	-	-	-
4.39	612	Not identified	*Liangia sinensis*	NR_173887.1	98%	96.37%
			Samsoniella hepiali	NR_160318.1	98%	96.01%
4.21	593	Not identified	*Fusarium ambrosium*	NR_173405.1	99%	98.14%
			Fusarium solani	NR_163531.1	100%	97.65%
			[Neocosmospora] rubicola	NR_154227.1	99%	97.63%
4.2	396	Not identified	-	-	-	-
1.2	396	Not identified	-	-	-	-
2.8	395	Not identified	-	-	-	-
4.9	643	Not identified	-	-	-	-
2.34	605	Not identified	*Aureobasidium melanogenum*	NR_159598.1	97%	99.66%
			Aureobasidium leucospermi	NR_156246.1	93%	98.77%
			Kabatiella bupleuri	NR_121524.1	92%	97.35%
